# Editorial: Physiological events associated with pesticide-resistance

**DOI:** 10.3389/fphys.2023.1286971

**Published:** 2023-10-18

**Authors:** Xun Zhu, Feng Liu, Zhiguang Yuchi, Xin Yang, Muhammad Shakeel

**Affiliations:** ^1^ State Key Laboratory for Biology of Plant Diseases and Insect Pests, Institute of Plant Protection, Chinese Academy of Agricultural Sciences, Beijing, China; ^2^ Institute of Infectious Disease, Shenzhen Bay Laboratory, Shenzhen, China; ^3^ Tianjin Key Laboratory for Modern Drug Delivery and High-Efficiency, Collaborative Innovation Center of Chemical Science and Engineering, School of Pharmaceutical Science and Technology, Tianjin University, Tianjin, China; ^4^ State Key Laboratory of Vegetable Biobreeding, Institute of Vegetables and Flowers, Chinese Academy of Agricultural Sciences, Beijing, China; ^5^ Key Laboratory of Bio-Pesticide Innovation and Application of Guangdong Province, College of Plant Protection, South China Agricultural University, Guangzhou, China

**Keywords:** agrochemical, pesticide resistance, pest management, resistance mechanisms, physiological changes

In agriculture, pesticide input is essential for ensuring food production. However, extensive use of pesticides in agricultural practices has led to increasing pesticide resistance. The evolution of resistance is vital for species’ survival under the selection pressure of pesticides, and resistance is considered a major obstacle in the development and application of pesticides. Therefore, understanding the resistance mechanisms is a prerequisite for managing it.

The goal of the present Research Topic on the “*Physiological Events Associated with Pesticide-Resistance*” in Frontiers in Physiology is to improve our understanding of the physiological responses of host plants and pests associated with pesticide resistance, reduce the risks related to pesticide use, and promote the development of new strategies to overcome the emerging resistance crisis. This Research Topic has collected five scientific contributions from highly qualified research groups focusing on resistance mechanisms and coping strategies.

At present, the molecular mechanisms underlying resistance generation and maintenance are not yet fully understood because of a lack of comprehensive structural and functional studies. Biological invasion is another major problem, besides resistance to agricultural development. Therefore, it is necessary to have a complete knowledge of the existing resistance mechanisms of invasive species to manage them. Based on previous studies, Siddiqui et al. summarized the resistance mechanisms of invasive species, including behavioral resistance, fitness cost, penetration resistance, target-site resistance, metabolic resistance, and resistance-inducing operational factors. Changes in the activity of metabolism enzymes in resistant species are of the utmost importance and are the most extensively researched Research Topic, specifically including esterases, glutathione S-transferase, cytochromes p450 monooxygenase, and hydrolysis.

Transcriptome analysis is a common technique for drug resistance studies. It reveals some causes of resistance by analyzing differentially expressed genes (DEGs). Siddiqui et al. collected *Solenopsis invicta* populations from 5 different cities in Guangdong, China to investigate the development of resistance to beta-cypermethrin and fipronil, and found that the Guangzhou population had the highest resistance against both insecticides. The enzymatic activities of acetylcholinesterase, carboxylases, and glutathione S-transferases were significantly elevated with increasing beta-cypermethrin and fipronil concentrations. The transcriptomic analysis uncovered 117 DEGs in beta-cypermethrin treatment (39 upregulated and 78 downregulated) and 109 DEGs in fipronil treatment (33 upregulated and 76 downregulated). Kyoto Encyclopedia of Genes and Genomes (KEGG) analysis showed that the genes associated with insecticide resistance were significantly enriched in metabolic pathways. Thus, detoxification enzymes play a significant role in insecticide detoxification and resistance development.

Insect symbiotic microorganisms co-evolve with their hosts over a long period, providing survival advantages to the host. Insect guts supply unique habitats for microbial colonization, and gut bacteria may serve hosts with a variety of useful services including insecticide resistance resulting from enhanced metabolic detoxification. Blanton et al. isolated a strain of bacteria from the gut of termites and cultured them continuously in a medium containing imidacloprid. They found that continuously cultured isolates grew in a range of gradually increasing concentrations of imidacloprid. After several generations of continuous exposure, the isolates grew significantly better than previous generations. These results suggest that the symbionts can rapidly adapt to increasing insecticide concentrations, which may contribute to the host’s resistance to insecticides.

As insect resistance has developed, it is urgent to find suitable management strategies, such as developing new pesticides or finding suitable ways to trap pests. Yin et al. investigated the important odor cues of *Apolygus lucorum* and *Adelphocoris suturalis* and found that tetradecane caused a strong EAG response in mirid bugs, while its derivatives, tetradecane and tetradecanoic acid, were also strongly attractive to them. However, the contents of tetradecane in the volatiles of common host plants were strikingly different. This may affect the olfactory responses of these two species of mirid bugs to different host plants. These results suggest that tetradecane plays a crucial role in the host selection of mirid bugs and can be used as a field bait to control these pests. Iftikhar et al. evaluated the toxicity of spirotetramat against adult apterous *B. brassicae* L. using the leaf dipping method. The sublethal concentrations (LC_5_ and LC_15_) and transgenerational effects of spirotetramat on population growth were estimated using the age-stage, two-sex life table theory. Sublethal concentrations of spirotetramat reduced the adult longevity and fecundity of the parent generation (F0) and decreased pre-adult survival, adult longevity, and reproduction of the F1 generation. In addition, the population growth parameters, such as the intrinsic rate of increase *r*, finite rate of increase λ, and net reproductive rate R0 of the F1 generation were decreased in the spirotetramat-treated groups. These results indicate that sublethal concentrations of spirotetramat impair the survival of *B. brassicae*, which may be useful for implementing IPM programs in pest control.

Pesticide resistance has always been a hot Research Topic during the development of modern agriculture. These five impressive studies provide different perspectives on the physiological responses associated with resistance and coping strategies, aiming to provide a new prospect for the sustainable use of pesticides.

